# The aminoacyl-tRNA synthetases of *Drosophila melanogaster*

**DOI:** 10.1080/19336934.2015.1101196

**Published:** 2016-01-13

**Authors:** Jiongming Lu, Steven J Marygold, Walid H Gharib, Beat Suter

**Affiliations:** 1 Institute of Cell Biology; University of Bern; Bern, Switzerland; 2 FlyBase; Department of Genetics; University of Cambridge; Cambridge, UK; 3 Interfaculty Bioinformatics Unit; University of Bern; Bern, Switzerland

**Keywords:** aminoacyl-tRNA synthetase, Charcot-Marie-Tooth neuropathy, *Drosophila* gene family, multifunctional protein, translation

## Abstract

Aminoacyl-tRNA synthetases (aaRSs) ligate amino acids to their cognate tRNAs, allowing them to decode the triplet code during translation. Through different mechanisms aaRSs also perform several non-canonical functions in transcription, translation, apoptosis, angiogenesis and inflammation. *Drosophila* has become a preferred system to model human diseases caused by mutations in *aaRS* genes, to dissect effects of reduced translation or non-canonical activities, and to study aminoacylation and translational fidelity. However, the lack of a systematic annotation of this gene family has hampered such studies. Here, we report the identification of the entire set of *aaRS* genes in the fly genome and we predict their roles based on experimental evidence and/or orthology. Further, we propose a new, systematic and logical nomenclature for aaRSs. We also review the research conducted on *Drosophila* aaRSs to date. Together, our work provides the foundation for further research in the fly aaRS field.

## Introduction

**Figure 1.**
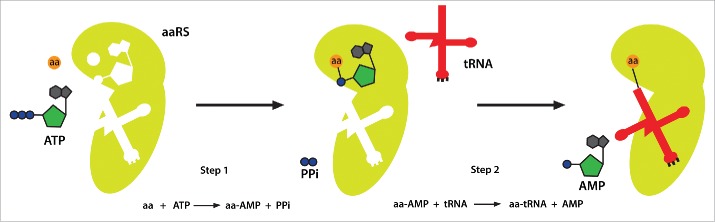
Aminoacyl-tRNA synthetase catalyzes a 2-step aminoacylation reaction. In the first step, the aaRS activates the substrate amino acid. By consuming an ATP it forms an aa-AMP intermediate. In the second step, the aa-AMP is transferred to the acceptor end of the cognate tRNA, generating an aa-tRNA that can be delivered to ribosomes for protein synthesis. aa, amino acid; aaRS, aminoacyl-tRNA synthetase; PPi, pyrophosphate.

Aminoacyl-tRNA synthetases (aaRSs) constitute an ancient family of enzymes that catalyze aminoacylation reactions by attaching amino acids to cognate tRNAs.^1,2^ The aminoacylation reaction is a 2-step process (**Fig. 1**). In the first step, the amino acid is activated by ATP to generate an aminoacyl-adenylate intermediate. In the second step, the activated amino acid is transferred to the 3′ end of the tRNA bearing the appropriate anticodon triplet that recognizes the corresponding codon in the mRNA.^3^ Such an aminoacylated tRNA is referred to as aa-tRNA and it can now be delivered to the ribosome for nascent polypeptide synthesis. Because aaRSs recognize specific amino acids and the corresponding tRNAs, they translate the nucleic acid language into the amino acid language and thereby decode the “second genetic code”.^4^ aaRSs are thus fundamental components of the protein synthesis process in all cells of all species in the 3 primary kingdoms of life.

There are 20 standard amino acids, and for each of them cells are expected to express at least one aaRS. Two different criteria may be used to categorize aaRSs. Based on their protein structure, class I aaRSs contain a characteristic Rossman fold catalytic domain and usually function as monomeric or dimeric proteins, while class II aaRSs contain 3 conserved motifs and are usually dimeric or tetrameric.^2,5^ Alternatively, aaRSs may be classified according to their subcellular sites of action: cytoplasmic, mitochondrial, or both cytoplasmic and mitochondrial (‘dual-localized’).^6,7^

Interest in aaRSs has grown in recent years for 2 major reasons. First, it has become apparent that aaRSs perform diverse non-canonical functions in addition to their roles in protein synthesis, including roles in regulation of transcription and translation, apoptosis, angiogenesis and inflammation.^8,9^ These additional functions are mainly achieved by recruitment of other protein complexes, acquisition of additional domains, or generation of novel protein fragments by alternative splicing or proteolysis.^8,10,11^ Second, genetic studies have revealed that mutations in many *aaRS* genes are associated with a wide variety of human syndromes and diseases.^7,12,13^ For example, mutations in 5 genes encoding cytoplasmic or dual-localized aaRSs have been identified in patients with (mainly dominantly inherited) peripheral neuropathies, while 9 mitochondrial *aaRS* loci have been implicated in heterogeneous recessive disorders.^12^ In most cases it is not known how *aaRS* mutations cause the disease phenotypes-whether through reduced translational activity, reduced aminoacylation accuracy or through a defect in a non-canonical function.

The powerful genetic tools available in *Drosophila melanogaster* offer tremendous potential to explore genotype – phenotype relationships, while the high evolutionary conservation of the tRNA aminoacylation reaction validates the modeling of aaRS-associated diseases in this system.^14,15^ However, the full complement of *Drosophila* aaRSs has not been accurately characterized to date, and as a result the annotations in the FlyBase database have been incomplete and in some cases incorrect. For example, prior to our recent study,^16^ the gene encoding mitochondrial PheRS was named as “*Aats-phe, phenylalanyl-tRNA synthetase*” (implying a cytoplasmic role) while the genes encoding the 2 subunits of the true cytoplasmic PheRS were unnamed. The lack of a comprehensive and consistent set of aaRS annotations in *D. melanogaster* potentially hampers understanding and research of these fundamental enzymes in this key model organism.

Here, we report our systematic analysis to identify and classify all aaRSs in *D. melanogaster*. In so doing, we propose a new nomenclature for *Drosophila* aaRS genes that is more explicit and consistent with that used in the wider field. In addition, we review the important aaRS studies that have been carried out in flies to date to illustrate how this model organism has already contributed to the field.

### Identification of *D. melanogaster* aminoacyl-tRNA synthetases

In a typical eukaryotic cell, there are cytoplasmic, mitochondrial, and dual-localized aaRSs.^7^ Because the number of standard amino acids is 20, the total number of aaRSs is therefore expected to be in the range of 20 to 40. We are aware of one previous study that attempted to identify the *D. melanogaster* aaRSs_17_ – this list of 20 different aaRSs, however, comprised a mixture of cytoplasmic and mitochondrial factors.

We began our own study of *D. melanogaster* aaRSs by searching FlyBase^18^ (FB2014_06) for genes with the prefix used in the database for this set of genes, namely ‘*Aats-*’, for ‘*Aminoacyl-tRNA synthetase*’. Only 22 *aaRS* genes were found by this approach (**Table S1**), suggesting that additional *aaRS* genes remained to be identified. Furthermore, reference to the location of the enzyme was inconsistent or missing in several gene names: 7 of the 22 named genes encode mitochondrial aaRSs, but this was indicated in only 2 cases; the names of the other 5 mitochondrial *aaRS* genes did not contain location information and would therefore be wrongly considered to be cytoplasmic (or dual-localized), particularly as the true cytoplasmic form was unnamed in each case (see below).

In order to identify the full complement of fly genes encoding aaRSs, we used the well-characterized set of human aaRS proteins (obtained from the HGNC database^19^) to search for matching *D. melanogaster* polypeptides in FlyBase (FB2014_06, Dmel Annotation Release 6.03) using BLASTP. We also examined ortholog predictions housed within FlyBase and the HGNC databases, and searched for genes annotated with relevant Gene Ontology terms and InterPro domains. The results are summarized in **Table 1**. We found that 35 genes in the fly nuclear genome encode 34 aaRS enzymes: 15 aaRSs are predicted to act exclusively in the cytoplasm, 15 in the mitochondria, and 4 are dual-localized. The reason for the gene count being one more than the aaRS count is because the cytoplasmic phenylalanyl-tRNA synthetase comprises 2 subunits encoded by 2 separate genes.^16^ The reason for finding 19 (and not 20) aaRSs that act in the cytoplasm is because the cytoplasmic glutamyl-prolyl-tRNA synthetase (GluProRS) loads both Glu and Pro to their cognate tRNAs.^20^ Finally the explanation for finding 19 (and not 20) aaRSs that function in mitochondria is that there is no mitochondrial glutaminyl-tRNA synthetase (GlnRS; discussed below). It is also worth noting that *CG10802, CG8097* and *Slimp* (*CG31133*), encode proteins containing domains associated with alanyl-, arginyl- and seryl-tRNA synthetase activity, respectively (**Table S2**). However, their overall similarities to the canonical human and *Drosophila* proteins are relatively low, and it is known that Slimp lacks aminoacylation activity.^21^ These three genes are therefore not included in **Table 1** and were not considered further.

**Table 1.** *D. melanogaster* aminoacyl-tRNA synthetases

Amino Acid	New Symbol	New Full Name	CG number	Localization	Ppt	Human Symbol / Identity	Ref.
Ala	*AlaRS*	*Alanyl-tRNA synthetase*	*CG13391*	C	1	AARS / 60.4%	
	*AlaRS-m*	*Alanyl-tRNA synthetase, mitochondrial*	*CG4633*	M	1	AARS2 / 30.6%	38, 39
Arg	*ArgRS*	*Arginyl-tRNA synthetase*	*CG9020*	C	1	RARS / 56.0%	
	*ArgRS-m*	*Arginyl-tRNA synthetase, mitochondrial*	*CG10092*	M	1	RARS2 / 32.5%	35
Asn	*AsnRS*	*Asparaginyl-tRNA synthetase*	*CG10687*	C	1	NARS / 70.6%	
	*AsnRS-m*	*Asparaginyl-tRNA synthetase, mitochondrial*	*CG6796*	M	1	NARS2 / 37.4%	
Asp	*AspRS*	*Aspartyl-tRNA synthetase*	*CG3821*	C	1	DARS / 63.3%	26
	*AspRS-m*	*Aspartyl-tRNA synthetase, mitochondrial*	*CG31739*	M	1	DARS2 / 25.8%	
Cys	*CysRS*	*Cysteinyl-tRNA synthetase*	*CG8431*	C	1	CARS / 57.5%	
	*CysRS-m*	*Cysteinyl-tRNA synthetase, mitochondrial*	*CG8257*	M	1	CARS2 / 36.1%	
Gln^*^	*GlnRS*	*Glutaminyl-tRNA synthetase*	*CG10506*	C	1	QARS / 57.2%	
Glu	GluProRS^**^	*Glutamyl-prolyl-tRNA synthetase*	*CG5394*	C	2	EPRS / 49.3%	20, 23
	*GluRS-m*	*Glutamyl-tRNA synthetase, mitochondrial*	*CG4573*	M	1	EARS2 / 43.5%	
Gly	*GlyRS*	*Glycyl-tRNA synthetase*	*CG6778*	C+M	2	GARS / 54.2%	43, 49-51
His	*HisRS*	*Histidyl-tRNA synthetase*	*CG6335*	C+M	3	HARS / 63.8% HARS2 / 54.3%	
Ile	*IleRS*	*Isoleucyl-tRNA synthetase*	*CG11471*	C	1	IARS / 50.5%	
	*IleRS-m*	*Isoleucyl-tRNA synthetase, mitochondrial*	*CG5414*	M	1	IARS2 / 36.5%	
Leu	*LeuRS*	*Leucyl-tRNA synthetase*	*CG33123*	C	1	LARS / 58.1%	
	*LeuRS-m*	*Leucyl-tRNA synthetase, mitochondrial*	*CG7479*	M	1	LARS2 / 38.6%	
Lys	*LysRS*	*Lysyl-tRNA synthetase*	*CG12141*	C+M	2	KARS / 63.8%	44
Met	*MetRS*	*Methionyl-tRNA synthetase*	*CG15100*	C	1	MARS / 41.9%	
	*MetRS-m*	*Methionyl-tRNA synthetase, mitochondrial*	*CG31322*	M	1	MARS2 / 40.4%	36
Phe	α*-PheRS*	*Phenylalanyl-tRNA synthetase*, α*-subunit*	*CG2263*	C	1	FARSA / 60.5%	16
	β*-PheRS*	*Phenylalanyl-tRNA synthetase*, β*-subunit*	*CG5706*	C	1	FARSB / 62.0%	16
	*PheRS-m*	*Phenylalanyl-tRNA synthetase, mitochondrial*	*CG13348*	M	1	FARS2 / 46.5%	34
Pro	GluProRS^**^	*Glutamyl-prolyl-tRNA synthetase*	*CG5394*	C	2	EPRS / 49.3%	20, 23
	*ProRS-m*	*Prolyl-tRNA synthetase, mitochondrial*	*CG12186*	M	2	PARS2 / 36.9%	
Ser	*SerRS*	*Seryl-tRNA synthetase*	*CG17259*	C	1	SARS / 66.2%	21
	*SerRS-m*	*Seryl-tRNA synthetase, mitochondrial*	*CG4938*	M	1	SARS2 / 28.4%	21, 37
Thr	*ThrRS*	*Threonyl-tRNA synthetase*	*CG5353*	C+M	2	TARS / 73.6% TARS2 / 50.6%	
Trp	*TrpRS*	*Tryptophanyl-tRNA synthetase*	*CG9735*	C	1	WARS / 55.0%	17
	*TrpRS-m*	*Tryptophanyl-tRNA synthetase, mitochondrial*	*CG7441*	M	1	WARS2 / 26.6%	
Tyr	*TyrRS*	*Tyrosyl-tRNA synthetase*	*CG4561*	C	1	YARS / 66.5%	15, 47
	*TyrRS-m*	*Tyrosyl-tRNA synthetase, mitochondrial*	*CG16912*	M	1	YARS2 / 45.2%	40-42
Val	*ValRS*	*Valyl-tRNA synthetase*	*CG4062*	C	2	VARS / 49.5%	
	*ValRS-m*	*Valyl-tRNA synthetase, mitochondrial*	*CG5660*	M	1	VARS2 / 32.9%	

* There is no mitochondrial GlnRS (see text for details).

** The GluProRS enzyme is a bi-functional enzyme, and thus appears twice in this table. Localization: C = cytoplasmic, M = mitochondrial; dual-localized aaRSs are annotated with ‘C+M’. Ppt: predicted unique polypeptides. Identity: amino acid identity between the longest isoforms of *D. melanogaster* and *H. sapiens* aaRSs (calculated using the CLUSTALO program).

We propose a unified *Drosophila* nomenclature for aaRSs that discriminates between the cytoplasmic and mitochondrial enzymes, rather than one that describes their biochemical and structural properties. This makes sense for work in a system that has a strong emphasis on functional studies. Furthermore, this nomenclature is widely used within the aaRS field. Thus we add a ‘-m’ suffix to the symbols of genes encoding the mitochondrial aaRSs. We also suggest that the ‘*Aats-x*’ format previously used for aaRS genes in FlyBase is replaced with the more common ‘*xRS*‘ format, where *x* indicates the relevant amino acid. Finally, we recommend using the 3-letter, rather than the single letter, amino acid code because this is more explicit and more easily recognized as an amino acid when followed by ‘RS’ in the same word and, again, it is a common convention in the field. With this nomenclature, the gene symbol for the cytoplasmic tyrosyl-tRNA synthetase is ‘*TyrRS*’, while ‘*TyrRS-m’* is the designation for the gene encoding the mitochondrial tyrosyl-tRNA synthetase. While it might also make sense to add a distinguishing suffix to the symbols of the dual-localized enzymes and genes, we have opted to name them the same way as the cytoplasmic ones to keep the symbols simple and short. The symbols and names of all aaRSs following this proposed nomenclature are shown in full in **Table 1**.

In the following parts, we will analyze the 3 groups of aaRSs separately and will review the published work on them.

### Cytoplasmic aminoacyl-tRNA synthetases

Cytoplasmic aaRSs charge tRNAs with cognate amino acids in the cytoplasm. Some of these aaRSs are also able to translocate to the nucleus and aminoacylation can also take place in this compartment.^22^ There are 16 genes encoding 15 cytoplasmic aaRSs in *D. melanogaster* and these are able to charge 16 different amino acids (**Table 1**). As mentioned, the discrepancies in these figures are explained by the *GluProRS* gene encoding a protein with 2 enzymatic activities, and by the PheRS enzyme consisting of 2 different subunits encoded by 2 distinct genes. While cytoplasmic *aaRS* genes generally encode a single, unique polypeptide, it is noteworthy that *GluProRS* and *ValRS*, respectively, encode each 2 different polypeptides, generated by alternative promoter usage and alternative splicing, respectively (FlyBase).

The bifunctionality of GluProRS is unique among all aaRSs and it has been well studied in various systems, including flies.^20,23^ In bacteria and archaea, 2 distinct genes encode GluRS and ProRS, and it seems that a gene fusion event occurred during the evolution of metazoa.^24^ The GluProRS protein is composed of 3 domains, the N-terminal domain with Glu-catalyzing activity, the C-terminal domain with Pro-catalyzing activity, and the central domain with repeated motifs. In *Drosophila*, the *GluProRS* gene encodes 2 polypeptides, the full-length protein and the C-terminal short protein. Their expression seems to be controlled by different promoters and probably distinct transcriptional regulators. The full-length protein is expressed throughout development, while the C-terminal short protein is especially abundant in 5–10 hours old embryos.^23^ Interestingly, the C-terminal short protein is functional in Pro-tRNA aminoacylation *in vivo*,^23^ thereby providing a second way to generate Pro-tRNA in the cytoplasm.

An interesting feature of cytoplasmic aaRSs in higher eukaryotes (including flies) is that 8 aaRSs, together with 3 non-enzymatic factors, form a ‘multi-synthetase complex’ (MSC).^9,20,25^ The functional significance of the MSC is unclear, but the auxiliary factors are thought to be responsive to diverse signal transduction pathways and thus provide a mechanism to coordinate protein synthesis with other biological processes.^9^ In flies, the auxiliary factors are encoded by the *CG8235, CG12304*, and *CG30185* genes, and we propose that these are named *AIMP1, AIMP2* and *AIMP3* (aminoacyl-tRNA synthetase-interacting multifunctional proteins 1, 2 and 3), respectively, to match the nomenclature used in the wider field (Supplementary **Table S2**).

Several cytoplasmic aaRSs have been discovered in different genetic screens in flies. *Aspartyl-tRNA synthetase* (*AspRS*) was independently identified in screens for *Sex-lethal* dosage-sensitive modifiers^26^ and for mutants defective in larval growth.^27^ *Tryptophanyl-tRNA synthetase* (*TrpRS*) was identified in a screen for genes expressed in the embryonic salivary gland,^17^ while mutations in several different cytoplasmic aaRSs were found to increase lysosomal activity.^28^ In each case, the specificity and precise function of the aaRS enzyme(s) involved remain to be characterized.

Work from our group has characterized the importance of aminoacylation fidelity *in vivo* by exploring the ‘double-sieving’ function of PheRS in *Drosophila*.^16^ The first sieve — amino-acid recognition — serves to exclude most non-cognate amino acids; the second sieve — amino-acid editing — is capable of correcting aminoacylation errors. Both sieves are important and double-sieving-defective mutations in *PheRS* result in misacylation by non-cognate Tyr and protein mistranslation, leading to many defects, including ER stress, neuronal cell apoptosis, impaired locomotive performance, reduced lifespan, and decreased organ size. This work demonstrates how malfunctioning of aaRSs at the molecular level can cause a range of phenotypes at the cellular and organismal levels.

### Mitochondrial aminoacyl-tRNA synthetases

**Figure 2.**
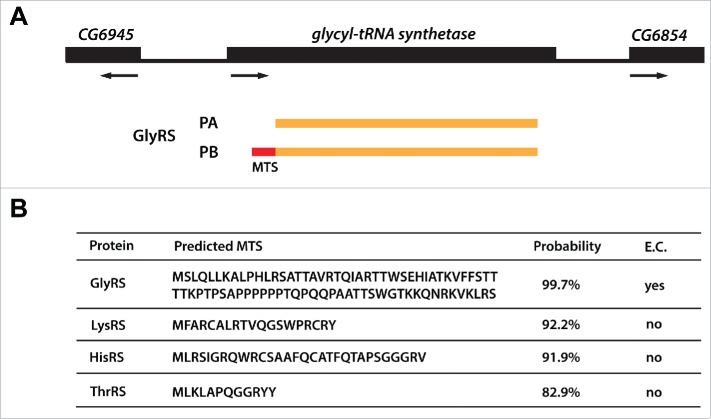
Dual-localized aaRSs in *D. melanogaster*. (**A**) GlyRS is shown as an example of dual-localized aaRS. It encodes 2 polypeptides, PA and PB. PB contains an extra N-terminal mitochondrial targeting sequence (MTS; in red) that can be used for its import into mitochondria. (**B**) The four dual-localized aaRSs with their predicted MTS and probabilities of mitochondrial localization. The analysis was performed by Mitoprot.^29^ E.C., experimentally confirmed.

Mitochondrial aaRSs are required for protein translation in this organelle and are thought to have a bacterial origin. In eukaryotic cells, they are encoded by nuclear genes and, after being expressed, they are imported into mitochondria with the guidance of a mitochondrial targeting sequence (MTS^29^; **Fig. 2**) There are 15 genes coding for 15 mitochondrial aaRSs in *D. melanogaster* (**Table 1**). In contrast to the situation in the cytoplasm, the mitochondrial PheRS consists of only one polypeptide encoded by a single gene while 2 separate genes code for distinct mitochondrial GluRS and ProRS enzymes. Furthermore, only *ProRS-m* is annotated to encode more than one polypeptide (FlyBase), though the function of the shorter protein isoform is unknown. We were unable to identify a *GlnRS-m* gene in our analysis, though this is consistent with a lack of GlnRS activity in all chloroplasts and mitochondria examined.^30,31^ In these organelles, Gln-tRNA is generated by mischarging a tRNA^Gln^ with Glu and converting Glu to Gln via a heterotrimeric Glu-tRNA^Gln^ amidotransferase (Gat).^31,32^ In *D. melanogaster*, the 3 subunits of this complex are encoded by the *GatA*,^33^ *CG5463* and *CG33649* genes – we propose to name the latter two *GatB* and *GatC*, respectively (**Table S2**).

*Drosophila* mitochondrial aaRSs have received a similar degree of attention in the published literature as their cytoplasmic counterparts. The sequence and structure of the fly PheRS-m was described in a comparative study with the human enzyme.^34^ *ArgRS-m* was identified in a genetic screen for nuclear-encoded genes with mitochondrial function.^35^ Its mitochondrial localization was confirmed in this study by using a GFP fusion protein. *MetRS-m* was identified in a screen for genes required for neuronal survival and function.^36^ Mutant flies were characterized and found to exhibit defects in mitochondrial function and cell proliferation. The function of *SerRS-m* was analyzed through an RNAi approach.^37^ This was shown to specifically reduce serylation of mitochondrial tRNAs, resulting in defective mitochondrial translation and function. AlaRS-m was studied to address how it distinguishes mitochondrial tRNA^Ala^ from the cytoplasmic tRNA^Ala^.^38,39^ Another series of experiments explored the compatibility between mitochondria-encoded tRNAs and their nucleus-encoded mitochondrial aaRSs.^40-42^ While individual mutations in a mitochondrial *tRNA*^*Tyr*^ gene and a mitochondrial *TyrRS-m* showed few phenotypic effects on their own, these mutations caused severe phenotypes coupled with reduced mitochondrial function when combined in the same fly.

### Dual-localized aminoacyl-tRNA synthetases

Dual-localized aaRSs are encoded by single genes and perform aminoacylation of tRNAs in the cytoplasmic and the mitochondrial compartments. Our database searches uncovered 4 candidate dual-localized aaRSs in *D. melanogaster* - GlyRS, LysRS, HisRS, and ThrRS (**Table 1**). Significantly, and in contrast to the genes in the other 2 groups, each of these aaRS genes encodes at least 2 polypeptides. As the mitochondrial version needs an MTS, it is possible that the shorter polypeptide corresponds to the cytoplasmic version and the longer one to the mitochondrial isoform. Indeed, this has been experimentally confirmed for GlyRS (**Fig. 2A**)^43^ and was suggested for LysRS.^44^ We analyzed the 2 other aaRSs using Mitoprot, a prediction tool for mitochondrial targeting sequences.^29^ Indeed, *HisRS* and *ThrRS* each encode at least one longer polypeptide with high probability of mitochondrial localization (**Fig. 2B**), strongly suggesting that these enzymes do indeed function as dual-localized aaRSs in *Drosophila*.

Despite the high conservation of these enzymes and their function through evolution, we noticed that the sets of fly and human dual-localized aaRS genes are not identical. Humans contain only 2 dual-localized *aaRS* genes, *GlyRS* and *LysRS*, while flies additionally have *HisRS* and *ThrRS*. This difference needs to be considered when modeling human diseases related to these 2 genes in flies.

**Figure 3.**
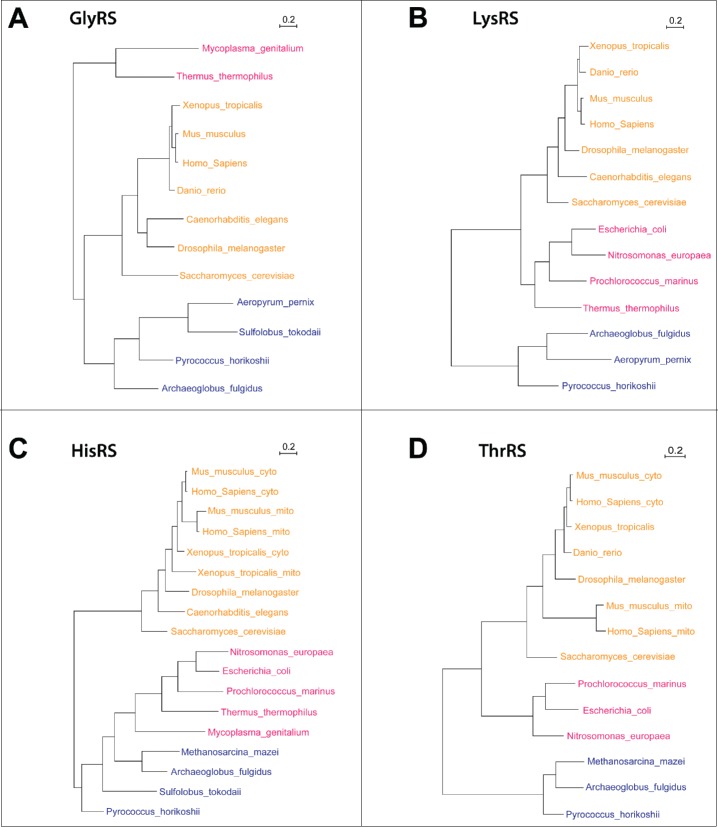
Phylogenetic analysis of 4 dual-localized aaRSs. Protein sequences of common eukaryotes, archaea, and bacteria were obtained from different databases (UniProt, Ensembl, HGNC, FlyBase, Xenbase, WormBase), and also by searching with BLAST. The sequences were aligned using Pagan,^54^ followed by TrimAl analysis,^55^ discarding the poorly aligned columns with the threshold of 60%. The treated multiple sequence alignments were used to generate the 4 gene trees using PhyML^56^; for topology searches we chose the best out of the NNI and PhyML-Subtree-Pruning-Regrafting (SPR) methods.^57,58^ All parameters were optimized, i.e., tree topology, branch length and the substitution rate. The number of bootstrap replicates was set to 5. Eukaryotes are shown in yellow, archaea in blue, and bacteria in red. The scale bar stands for the number of substitutions per site.

To obtain a better understanding of how these 4 enzymes evolved, we performed phylogenetic analyses with sequences from various eukaryotic species (including flies and human), bacteria and archaea (**Fig. 3**). All eukaryotic species analyzed contain a single *GlyRS* gene, which is more closely related to the one from archaea than to the bacterial one, suggesting that it originated from the cytoplasmic aminoacyl-tRNA synthetases. LysRS is also encoded by a single gene. However, the eukaryotic LysRS is closer to the bacterial ones, suggesting that it originated from a mitochondrial gene. Interestingly, lower eukaryotic species contain a single *HisRS* gene, just like bacteria and archaea, while vertebrates (higher eukaryotes) have 2 separate genes, demonstrating that splitting up their function into 2 separate genes and enzymes was beneficial to vertebrates. For *ThrRS* the available data suggest that lower eukaryotes may contain only one gene type that was derived from the bacterial/mitochondrial one. The phylogenetic tree then further suggests that there was a subsequent split into 2 types, but that this split was only maintained in some of the analyzed lineages, in the higher vertebrates (mammals). Clearly, more data points are needed to ascertain the apparently rather complex evolution of the ThrRS sequences.

GlyRS is the only dual-localized aaRS that has been studied experimentally in *Drosophila*. It was initially identified in a mosaic forward genetic screen for genes having cell-autonomous functions in dendritic and axonal development.^43^ While the cytoplasmic function of GlyRS was found to be required for terminal arborization of both dendrites and axons during development, the mitochondrial function is preferentially required for the maintenance of dendritic terminals in adults.

### *Drosophila* as a model for aaRS-associated human diseases

Mutations in multiple aaRSs have been implicated in several different human diseases, though the mechanistic details are obscure in most cases.^7^ Researchers have begun to use the power and efficiency of *Drosophila* genetics to model some of these diseases, and in so doing more readily investigate their molecular and cellular basis.

Charcot-Marie-Tooth (CMT) neuropathies affect the peripheral nervous system and are associated with axonal degeneration, distal muscle wasting and progressive motor impairment.^45^ Mutations in the human *YARS* gene, encoding the cytoplasmic TyrRS, cause dominant-intermediate CMT type C (DI-CMTC).^46^ Transgenic expression of either human or *Drosophila TyrRS* bearing disease-associated mutations in flies recapitulated several hallmarks of the human pathology, including progressive decreases in motor performance and axonal degeneration.^15,47^ By virtue of studying these effects in flies, the authors were able to conclude that the disease phenotypes are not caused by reduced aminoacylation activity, but are more likely due to a gain-of-function alteration of the mutant TyrRS or interference with a non-canonical function.^47^ This *Drosophila* disease model was also demonstrated to be a useful and rapid platform for screening the pathogenicity of novel candidate *YARS* mutations.^15^

Mutations in a second human aaRS gene, *GARS* (encoding the dual-localized GlyRS enzyme), cause a different CMT subtype, CMT type 2D (CMT2D).^48^ An initial study in flies found that loss-of-function mutations in the native *Drosophila GlyRS* gene resulted in neuronal phenotypes consistent with CMT2D symptoms in humans, although transgenic expression of disease-associated *GARS* mutations in neuronal clones had no morphological effect.^43^ A recent study generated a more realistic *Drosophila* model for CMT2D through ubiquitous or pan-neuronal expression of fly *GlyRS* transgenes with alterations equivalent to those of pathogenic *GARS* mutations. These transgenic flies showed both morphological and behavioral phenotypes that recapitulated the human disease.^49^ Significantly, these phenotypes were observed for disease-associated *GlyRS* mutants that maintained aminoacylation activity, suggesting that CMT2D is the result of a toxic, neomorphic activity, similar to the conclusion from the DI-CMTC model. A subsequent study confirmed these observations, and further suggested that the gain-of-function effects have a non-cell autonomous contribution.^50^ Other recent work has generated a complementary *Drosophila* model of CMT2D in flies through expression of human *GARS* transgenes harboring disease-associated mutations.^51^ The disease-relevant phenotypes were again found not to correlate with reduced aminoacylation activity of the enzyme. Nevertheless, a marked decrease in global protein synthesis in motor and sensory neurons was observed in the transgenic flies, suggesting that the mutant enzymes inhibit translation through a cell autonomous mechanism independent of their aminoacylation function. Interestingly, expression of DI-CMTC-associated *YARS* mutants also resulted in translation inhibition in this assay.^51^ This finding, together with the facts that the phenotypes of the fly models of both CMT subtypes are similar and share common genetic modifiers,^49^ suggests that a common mechanism may underlie both YARS- and GARS-associated CMT neuropathies.

Fly *MetRS-m* was identified in a screen for genes required for neuronal survival and function.^36^ Mutant flies exhibited defects in mitochondrial function, cell proliferation and age-dependent retinal and muscle degeneration. Remarkably, these findings led to the discovery that mutations in the orthologous human gene, *MARS2*, are responsible for the neurodegenerative disease Autosomal Recessive Spastic Ataxia with Leukoencephalopathy (ARSAL). Similar to flies, cells from ARSAL patients showed aberrant mitochondrial function and proliferation. This study also reported that treatment with antioxidants could suppress the fly mutant phenotypes, indicating a possible treatment for the human disease.

A different study utilized RNAi to target *SerRS-m* to produce a fly model of human mitochondrial aminoacylation pathologies in general and mitochondrial serylation defects in particular.^37^ For example, the fly phenotypes reproduce traits seen in MELAS (mitochondrial encephalomyopathy, lactic acidosis and stroke-like episodes) or MERRF (myoclonic epilepsy with ragged red fibers), as well as HUPRA syndrome (hyperuricemia, pulmonary hypertension, renal failure in infancy and alkalosis), the latter of which is caused by a mutation in the orthologous *SARS2* gene. Furthermore, it was found that antioxidant treatment ameliorated the phenotypes resulting from *SerRS-m* silencing,^37^ similar to the *MetRS-m* study.^36^

Finally, *Drosophila TyrRS-m* has been studied as a general model for human mitochondrial diseases stemming from an incompatibility between the nuclear-encoded aaRSs and mitochondrially-encoded tRNAs.^42^ In this model, a mutation in *TyrRS-m* resulted in defective mitochondrial dysfunction and locomotor defects, though the severity varied across different genetic backgrounds and traits. These context-dependent phenotypes mirror the symptoms of the MLASA syndrome (mitochondrial myopathy, lactic acidosis and sideroblastic anemia) that results from mutations in the orthologous human gene *YARS2*.

In summary, several different aaRS-associated human diseases have so far been effectively modeled in *Drosophila* using a variety of genetic techniques. These approaches have generated a number of clinically important conclusions, including insights into etiology of CMT^15,47,49-51^ and the discovery of the underlying cause of ARSAL.^36^ Moreover, some of these studies have isolated modifiers of the disease model,^36,37,49^ demonstrating a further advantage of using the *Drosophila* system.

## Conclusion

Most studies of aaRS biology in *Drosophila* to date have either investigated their canonical functions or have used mutant genotypes to produce models of human diseases linked to aaRS dysfunction. In addition, several aaRSs have been identified in diverse genetic screens, though their precise role in these conditions remains unclear. Notably, the non-canonical roles of aaRSs that have been described in other systems have so far received sparse attention in *Drosophila*, while many aaRS-associated human diseases have yet to be modeled in flies. Based on these considerations we predict an increase in such studies in the near future. The systematic identification and logical naming of the *D. melanogaster* aaRSs presented here, together with our literature survey, will aid all these lines of investigation, and thereby facilitate further discoveries into both the normal and aberrant mechanisms of action of these essential and fascinating enzymes.
