# Supplementary material for: The aminoacyl-tRNA synthetases of Drosophila melanogaster
Source: Fly (Austin). 2016 Jan 13;9(2):53–61. doi: 10.1080/19336934.2015.1101196 (PMC4826098; doi:10.1080/19336934.2015.1101196)
Supplement: 1101196_Supplemental_Material.zip [file kfly-09-02-1101196-s001.zip › Table S2.docx]

**Supplementary Table 2: Aminoacyl-tRNA synthetase related factors.**

The Table shows aaRS related genes that are reported in our study.

| **CG number** | **Symbol** | **Full Name / description** | **Ppt** | **Ref.** |
| --- | --- | --- | --- | --- |
| *CG8235* | *AIMP1* | *aaRS-interacting multifunctional protein 1* | 1 |  |
| *CG12304* | *AIMP2* | *aaRS-interacting multifunctional protein 2* | 2 |  |
| *CG30185* | *AIMP3* | *aaRS-interacting multifunctional protein 3* | 1 |  |
| *CG6007* | *GatA* | *glutamyl-tRNA amidotransferase subunit A* | 1 | 33 |
| *CG5463* | *GatB* | *glutamyl-tRNA amidotransferase subunit B* | 1 |  |
| *CG33649* | *GatC* | *glutamyl-tRNA amidotransferase subunit C* | 1 |  |
| *CG10802* |  | *AlaRS-like protein* | 2 |  |
| *CG8097* |  | *ArgRS-like protein* | 1 |  |
| *CG31133* | *Slimp* | *Seryl-tRNA synthetase-like insect mitochondrial protein* | 1 | 21 |

21. Guitart T, Leon Bernardo T, Sagales J, Stratmann T, Bernues J, Ribas de Pouplana L. New aminoacyl-tRNA synthetase-like protein in insecta with an essential mitochondrial function. J Biol Chem 2010; 285:38157-66.

33. Morris JZ, Bergman L, Kruyer A, Gertsberg M, Guigova A, Arias R, et al. Mutations in the Drosophila mitochondrial tRNA amidotransferase, bene/gatA, cause growth defects in mitotic and endoreplicating tissues. Genetics 2008; 178:979-87.
