# Supplementary material for: The aminoacyl-tRNA synthetases of Drosophila melanogaster
Source: Fly (Austin). 2016 Jan 13;9(2):53–61. doi: 10.1080/19336934.2015.1101196 (PMC4826098; doi:10.1080/19336934.2015.1101196)
Supplement: 1101196_Supplemental_Material.zip [file kfly-09-02-1101196-s001.zip › Table S1.doc]

**Supplementary Table 1:
*D. melanogaster* aminoacyl -tRNA synthetases annotated in FlyBase**

Table shows the results of searching for gene symbols with an ‘Aats-‘ prefix in FlyBase release FB2014_06. Asterisks (*) indicate genes now considered to encode mitochondrial aaRSs (this study).

| **Amino acid** | **Symbol** | **Full name** | **CG number** |
| --- | --- | --- | --- |
| Ala | *Aats-ala* | *Alanyl-tRNA synthetase* | CG13391 |
|  | *Aats-ala-m* | *mitochondrial alanyl-tRNA synthetase* | CG4633 |
| Arg | *Aats-arg* | *Arginyl-tRNA synthetase* | CG9020 |
| Asn | *Aats-asn* | *Asparaginyl-tRNA synthetase* | CG10687 |
| Asp | *Aats-asp* | *Aspartyl-tRNA synthetase* | CG3821 |
| Cys | *Aats-cys* | *Cysteinyl-tRNA synthetase* | CG8431 |
| Gln | *Aats-gln* | *Glutaminyl-tRNA synthetase* | CG10506 |
| Glu | *Aats-glupro* | *Glutamyl-prolyl-tRNA synthetase* | CG5394 |
| Gly | *Aats-gly* | *Glycyl-tRNA synthetase* | CG6778 |
| His | *Aats-his* | *Histidyl-tRNA synthetase* | CG6335 |
| Ile | *Aats-ile* | *Isoleucyl-tRNA synthetase* | CG11471 |
| Leu | *Aats-leu** | *Leucyl-tRNA synthetase* | CG7479 |
| Lys | *Aats-lys* | *Lysyl-tRNA synthetase* | CG12141 |
| Met | *Aats-met** | *Methionyl-tRNA synthetase* | CG31322 |
| Phe | *Aats-phe** | *Phenylalanyl-tRNA synthetase* | CG13348 |
| Pro | *Aats-pro** | *Prolyl-tRNA synthetase* | CG12186 |
| Ser | *Aats-ser** | *Seryl-tRNA synthetase* | CG4938 |
| Thr | *Aats-thr* | *Threonyl-tRNA synthetase* | CG5353 |
| Trp | *Aats-trp* | *Tryptophanyl-tRNA synthetase* | CG9735 |
| Tyr | *Aats-tyr* | *Tyrosyl-tRNA synthetase* | CG4561 |
|  | *Aats-tyr-m* | *mitochondrial tyrosyl-tRNA synthetase* | CG16912 |
| Val | *Aats-val* | *Valyl-tRNA synthetase* | CG4062 |
